# Changes in the illness perceptions of patients with rheumatoid arthritis over the first year of methotrexate therapy

**DOI:** 10.1093/rheumatology/keaa615

**Published:** 2020-11-14

**Authors:** James M Gwinnutt, Sam Norton, Kimme L Hyrich, Mark Lunt, Anne Barton, Lis Cordingley, Suzanne M M Verstappen, for the RAMS co-investigators

**Affiliations:** 1 Centre for Epidemiology Versus Arthritis, Centre for Musculoskeletal Research, Division of Musculoskeletal and Dermatological Sciences, School of Biological Sciences, Faculty of Biology, Medicine and Health, University of Manchester, Manchester Academic Health Science Centre, Manchester; 2 Health Psychology section, Institute of Psychiatry, Psychology and Neuroscience; 3 Department of Inflammation Biology, Faculty of Life Sciences and Medicine, King’s College London, London; 4 NIHR Manchester Biomedical Research Centre, Manchester University NHS Foundation Trust, Manchester Academic Health Science Centre and the; 5 Centre for Genetics and Genomics Versus Arthritis, Centre for Musculoskeletal Research, Division of Musculoskeletal and Dermatological Sciences, School of Biological Sciences, Faculty of Biology, Medicine and Health, University of Manchester, Manchester Academic Health Science Centre, Manchester, UK

**Keywords:** Rheumatoid Arthritis, Illness Perceptions, Health Psychology, Epidemiology, Disability

## Abstract

**Objectives:**

To describe the illness perceptions of patients with RA over the first year of MTX treatment, and the association between illness perceptions and outcomes.

**Methods:**

Data came from the Rheumatoid Arthritis Medication Study (RAMS), a UK multicentre cohort study of RA patients starting MTX for the first time. Patients were assessed at baseline, and at 6 and 12 months. Patients completed the Brief Illness Perception Questionnaire (B-IPQ) at each assessment, as well as other patient-reported outcomes (PROs). The inflammation score (2-component DAS28) was calculated. Subgroups of patients with similar trajectories across the eight (B-IPQ) items were identified using a latent class growth model. Predictors of group membership were identified using multinomial logistic regression. Associations between subgroups and PROs over follow-up were assessed using linear mixed models.

**Results:**

Three subgroups were identified in the analysis population (*N* = 1087): Positive illness perceptions (*N* = 322), Negative illness perceptions (*N* = 534) and Improvers (*N* = 231) who switched from negative to positive illness perceptions over follow-up. Baseline disability was associated with group membership [Positive *vs* Negative: relative risk ratio (RRR) 0.37, 95% CI: 0.25, 0.54; Improvers *vs* Negative: RRR 0.60, 95% CI: 0.43, 0.83], as were other PROs (pain, fatigue, anxiety, depression). The Negative group had worse disability, pain and fatigue over follow-up compared with the other groups, controlling for inflammation.

**Conclusion:**

Negative illness perceptions are associated with poor PROs over time. The Improvers subgroup illustrated that illness perceptions can change in RA. Illness perceptions represent a potential therapeutic target that should be assessed using randomized trials.

**Rheumatology key messages**

- Previously, research has only considered illness perceptions in rheumatoid arthritis (RA) at single time-points.
- This study demonstrated that illness perceptions changed longitudinally and were associated with outcomes in RA patients.
- Therefore, illness perceptions represent a potential therapeutic target in RA.

## Introduction

A biopsychosocial approach to disease recognizes that people’s experiences of long-term illness are mediated by biological, psychological and social factors [1]. RA is a chronic condition characterized by inflammation of joints, chronic disability and pain [2]. Outcomes in RA are related to biological factors, such as inflammation and serological markers [3–5], but also to psychosocial factors, such as higher helplessness and perceived stress [6, 7].

One set of psychological factors that are important in predicting outcomes in RA are patients’ illness perceptions. Leventhal *et al*'s Common-Sense Model of Self-Regulation states that people’s beliefs about their disease (i.e. illness perceptions) do not necessarily match their biological condition and yet are associated with outcomes [8]. A study of 134 Norwegian patients undergoing one or more weeks of rheumatology rehabilitation illustrated that poor illness perceptions were associated with worse pain, function and mental health 12 months later [9]. An Austrian cross-sectional study of 120 RA patients reported associations between stronger beliefs in the negative consequences of RA and physical health–related quality of life [10]. Another cross-sectional study of 230 patients with RA from the UK showed that illness perceptions were associated with increased psychological distress, independent of disease activity [11]. A study of 189 patients with established RA (mean disease duration = 12.6 years) from six UK hospitals used latent profile analysis to identify two subgroups of patients based on their illness perceptions. Those in the negative illness perception group had worse function, pain and distress than the positive illness group at 6 months, after adjustment for differences in clinical and demographic variables at baseline [12].

These studies assessed illness perceptions at a single time-point; however, illness perceptions have been shown to change over time in other musculoskeletal diseases. For instance, Bijsterbosch *et al.* showed that people with OA reported feeling that their OA was more chronic and less controllable at 6 years compared with baseline, but that they experienced fewer negative emotions because of their OA [13]. Indeed, Leventhal *et al*'s original model does not consider illness perceptions to be fixed for individuals. An individual’s perceptions evolve over time, based on the evolving symptoms of their disease and their attempts to cope with these symptoms [14]. A person’s perceptions can change considerably, particularly early in the disease course, while their mental schema of the illness (i.e. illness representation) is being formed [8]. Given that there are more effective treatments available for RA than OA, but also that RA symptoms typically fluctuate more over time than OA, one may expect different changes in illness perception over time in patients with RA compared with OA. However, at present there are few data on how illness perceptions change over time in patients with RA. Our hypothesis is that there are distinct subgroups of patients characterized by changes in their illness perceptions over 12 months, and that these illness perceptions are associated with outcomes. The aims of the current study were (i) to characterize how the illness perceptions of people with RA changed over 12 months following the initiation of MTX, (ii) to assess whether there are distinct groups of patients with similar trends in illness perceptions over time, (iii) to assess baseline factors predicting membership of these groups and (iv) to describe the outcomes for these groups over 12 months.

## Methods

Patients with RA who were about to start MTX treatment for the first time were recruited to the Rheumatoid Arthritis Medication Study (RAMS), a prospective cohort study based in the UK [15]. Patients were recruited from 38 centres across the UK from 2008. Patients were included in the analysis if they had illness perception data on at least one item at baseline (pre-treatment) and at either 6 or 12 months. Patients were excluded if they had >24 months of symptom duration at baseline (see Supplementary Fig. S1, available at *Rheumatology* online, for flow-diagram of exclusions). RAMS ethical approval was obtained from the National Research Ethics Service Central Manchester Research Ethics Committee (ref: 08/H1008/25), and all patients gave their written informed consent.

### Assessments

Demographics were collected at baseline, and patients completed questionnaires at baseline and at 6 and 12 months. Each patient’s socio-economic status was defined based on where they lived, using the Index of Multiple Deprivation 2010 (IMD) [16], coded as quintiles, with the lowest quintile being the most deprived. Patients completed the British version of the HAQ – Disability Index (HAQ-DI), a self-reported measure of functional disability [17]. Patients also completed pain and fatigue visual analogue scales (VASs) and the Hospital Anxiety and Depression Scale (HADS), calculating separate scores for the anxiety and depression subscales [18]. Blood samples were taken at each assessment and stored in –80°C freezers for analysis including: CRP (mg/l) level at each assessment and RF positivity (latex test) at baseline. The 2-component Disease Activity Score (DAS28-2C) was calculated at each assessment. This is a combination of swollen joint count and CRP, representing inflammation level rather than global disease activity [19]. The 4-component DAS28 (swollen and tender joint counts, CRP and patient global assessment) was also calculated [20, 21].

#### Illness perceptions

Illness perceptions were measured using the Brief Illness Perception Questionnaire (B-IPQ) [22], modified for patients with RA. This eight-item questionnaire was developed with the aim of being a very short yet valid measure of illness perceptions [23], ideal when large numbers of questionnaires are being delivered simultaneously. The items were developed by forming one question that best summarized each of the subscales on the Illness Perception Questionnaire – Revised. Five items represent cognitive illness representations (items 1–5), two represent emotional representations (items 6 and 8) and one represents illness comprehensibility (item 7). For B-IPQ items 1, 2, 5, 6 and 8, lower scores indicate more positive illness perceptions. For B-IPQ items 3, 4 and 7, higher scores indicate more positive illness perceptions. Therefore, throughout the paper, the term ‘improves’ indicates scores moving towards more positive illness perceptions, dependent on the anchoring of the item. For figures, items 3, 4 and 7 are reverse coded, so that for all items lower scores indicate more positive illness perceptions.

**Table 2:** Brief Illness Perception items over time for the total cohort and stratified by B-IPQ trajectory group

			Total cohort (*N* = 1087)		Negative (*N* = 534)		Positive (*N* = 322)		Improvers (*N* = 231)	
	#	B-IPQ item	Baseline, mean (s.d.)	12 months, mean (s.d.)	Baseline, mean (s.d.)	12 months, mean (s.d.)	Baseline, mean (s.d.)	12 months, mean (s.d.)	Baseline, mean (s.d.)	12 months, mean (s.d.)
Cognitive Illness representations	**1**	How much does arthritis affect your life? (10 = severely affects life)	5.6 (2.5)	4.3 (2.6)	7.0 (1.8)	5.5 (2.4)	2.8 (1.5)	2.9 (2.0)	6.3 (1.9)	3.5 (2.7)
	**2**	How long do you think your arthritis will continue? (10 = forever)	8.2 (2.5)	8.7 (2.4)	8.6 (2.1)	9.0 (1.8)	7.4 (3.1)	8.4 (2.8)	8.2 (2.3)	8.4 (2.8)
	**3**	How much control do you feel you have over your arthritis? (10 = extreme amount of control)	4.2 (2.7)	5.6 (2.7)	3.7 (2.6)	5.1 (2.6)	5.1 (2.8)	6.0 (2.7)	4.0 (2.6)	6.0 (2.9)
	**4**	How much do you think your treatment can help your arthritis? (10 = extremely helpful)	7.8 (1.9)	7.8 (2.2)	7.6 (1.9)	7.5 (2.2)	8.1 (1.9)	8.1 (2.2)	8.1 (1.8)	8.3 (2.3)
	**5**	How much do you experience symptoms from your illness? (10 = many severe symptoms)	6.2 (2.4)	4.7 (2.5)	7.4 (1.7)	5.7 (2.3)	3.7 (1.8)	3.7 (2.3)	6.7 (1.8)	4.1 (2.7)
Emotional representations	**6**	How concerned are you about your arthritis? (10 = extremely concerned)	7.9 (2.3)	6.1 (3.0)	8.8 (1.6)	7.3 (2.6)	6.1 (2.6)	4.8 (2.9)	8.3 (1.8)	5.3 (2.9)
Illness comprehensibility	**7**	How well do you feel you understand your arthritis? (10 = understand very clearly)	6.9 (2.4)	7.4 (2.3)	6.7 (2.5)	7.3 (2.4)	6.8 (2.5)	7.4 (2.3)	7.5 (2.2)	7.8 (2.4)
Emotional representations	**8**	How much does your arthritis affect you emotionally (e.g. does it make you angry, scared, upset or depressed)? (10 = extremely affected)	5.4 (2.9)	4.2 (3.0)	6.7 (2.4)	5.6 (2.8)	3.1 (2.4)	2.7 (2.4)	5.6 (2.9)	3.4 (2.8)

B-IPQ: Brief Illness Perception Questionnaire; *N*: number.

### Statistical analysis

The baseline characteristics of the patients and baseline and 12-month illness perception scores were summarized using descriptive statistics. A multivariate latent class growth model (including fixed and random effects) was used to identify groups of patients with similar illness perception trajectories over 12 months. The aim of the analysis was to categorize patients into distinct groups based on multiple continuous measures, assessed at multiple time-points [24]. Models with two to five latent classes with linear, quadratic and spline functions to model time were constructed, and the Bayesian Information Criterion was used to identify the best-fitting model. Models that identified trajectory groups containing <5% of the cohort were excluded. Individual items of the B-IPQ were compared between the trajectory groups using linear mixed models. Baseline independent predictors of group membership were assessed using multinomial logistic regression. Candidate predictors were age, gender, symptom duration, smoking status, DAS28-2C, HAQ, pain VAS, fatigue VAS, HADS depression, HADS anxiety, RF status and IMD. Given the relatively small numbers in the highest and lowest quintiles of IMD, IMD quintile was included in the logistic regression model as a continuous variable. The associations between trajectory group and HAQ, pain VAS and fatigue VAS over 12 months were assessed using linear mixed effects models, controlling for baseline age, gender and time-varying inflammation (DAS28-2C). Missing data were imputed using iterative chained equations. Statistical analyses were conducted using R, version 3.6.0 (packages: foreign, grid, gridExtra [25], lcmm [24], lme4 [26], mice [27], miceadds [28], nnet [29], tidyverse [30], and splines).

## Results

In total, 1087 patients were included in the analysis. The median age of the cohort was 61 years [interquartile range (IQR): 52, 70] at baseline, and 708 (65.1%) patients were women. Patients had moderate disease activity and disability at baseline [median (IQR): DAS28 = 4.1 (3.2, 5.1); HAQ = 1.0 (0.5, 1.5), Table 1]. The illness perceptions of the patients at baseline illustrated that patients perceived that their arthritis affected them moderately [mean (s.d.): B-IPQ1 = 5.6 (2.5), B-IPQ5 = 6.2 (2.4), B-IPQ8 = 5.4 (2.9)], that they had moderate control over their arthritis [mean (s.d.): B-IPQ3 = 4.2 (2.7)], that they thought their arthritis would last for a long time [mean (s.d.): B-IPQ2 = 8.2 (2.5)], but that treatment would be helpful [mean (s.d.): B-IPQ4 = 7.8 (1.9)] (Table 2). Furthermore, patients were highly concerned about their arthritis [mean (s.d.): B-IPQ6 = 7.9 (2.3)], but they understood their arthritis well [mean (s.d.): B-IPQ7 = 6.9 (2.4)].

**Table 1:** Baseline characteristics

	Total cohort (*N* = 1087)	Negative (*N* = 534)	Positive (*N* = 322)	Improvers (*N* = 231)
	Median (IQR)	Median (IQR)	Median (IQR)	Median (IQR)
Baseline characteristic	[% missing]	[% missing]	[% missing]	[% missing]
Age, years	61 (52, 70) [0]	60 (51, 69) [0]	63 (52, 70) [0]	62 (52, 69.5) [0]
Women, *N* (%)	708 (65.1) [0]	364 (68.2) [0]	195 (60.6) [0]	149 (64.5) [0]
Symptom duration, months	6.2 (3.7, 10.7) [0]	6.6 (4.0, 11.2) [0]	5.9 (3.6, 9.4) [0]	6.1 (3.5, 11.0) [0]
Smoking status, *N* (%)				
Never	441 (40.6)	211 (39.5)	130 (40.4)	100 (43.3)
Ex-smoker	457 (42.0)	218 (40.8)	140 (43.5)	99 (42.9)
Current	181 (16.7)	101 (18.9)	51 (15.8)	29 (12.6)
Missing	8 (0.7)	4 (0.7)	1 (0.3)	3 (1.3)
IMD quintiles				
1 (most deprived)	97 (8.9)	57 (10.7)	22 (6.8)	18 (7.8)
2	187 (17.2)	103 (19.3)	42 (13.0)	42 (18.2)
3	165 (15.2)	77 (14.4)	49 (15.2)	39 (16.9)
4	262 (24.1)	114 (21.3)	100 (31.1)	48 (20.8)
5 (least deprived)	249 (22.9)	111 (20.8)	72 (22.4)	66 (28.6)
Missing	127 (11.7)	72 (13.5)	37 (11.5)	18 (7.8)
DAS28	4.1 (3.2, 5.1) [3.9]	4.5 (3.6, 5.5) [3.2]	3.3 (2.7, 4.3) [5.3]	4.2 (3.4, 5.0) [3.5]
DAS28-2C	3.3 (2.2, 4.5) [3.4]	3.5 (2.5, 4.6) [2.6]	2.9 (1.7, 3.8) [5.0]	3.5 (2.5, 4.6) [3.0]
HAQ	1.0 (0.5, 1.5) [0.5]	1.4 (0.9, 1.9) [0.6]	0.4 (0.1, 0.9) [0.3]	1.0 (0.5, 1.5) [0.4]
VAS pain (0–100)	47 (24, 70) [2.5]	63 (43, 76) [3.2]	24 (12, 38) [1.6]	50 (25, 71) [2.2]
VAS fatigue (0–100)	50 (22, 72) [2.3]	65 (45, 78) [2.8]	22 (6, 43) [2.2]	49 (20, 70) [1.3]
HADS depression	5 (2, 8) [0.7]	7 (4, 10) [0.7]	2 (1, 5) [0.9]	4 (2, 7) [0.4]
HADS anxiety	6 (3, 9) [0.9]	7 (4, 10) [0.9]	4 (1, 6) [1.2]	5 (2, 8) [0.4]
RF status, *N* (%)				
Positive	586 (53.9)	279 (52.2)	178 (55.3)	129 (55.8)
Negative	302 (27.8)	156 (29.2)	80 (24.8)	66 (28.6)
Missing	199 (18.3)	99 (18.5)	64 (19.9)	36 (15.6)
Taking oral steroids, *N* (%)	260 (23.9) [0.3]	140 (26.2) [0.2]	71 (22.0) [0.3]	49 (21.2) [0.4]

DAS28: DAS 28; DAS28-2C: 2-component DAS; HADS: Hospital Anxiety and Depression Scale; IMD: Index of Multiple Deprivation 2010; IQR: interquartile range; *N*: number; VAS: visual analogue scale.

### B-IPQ change over 12 months

**Fig. 1:**
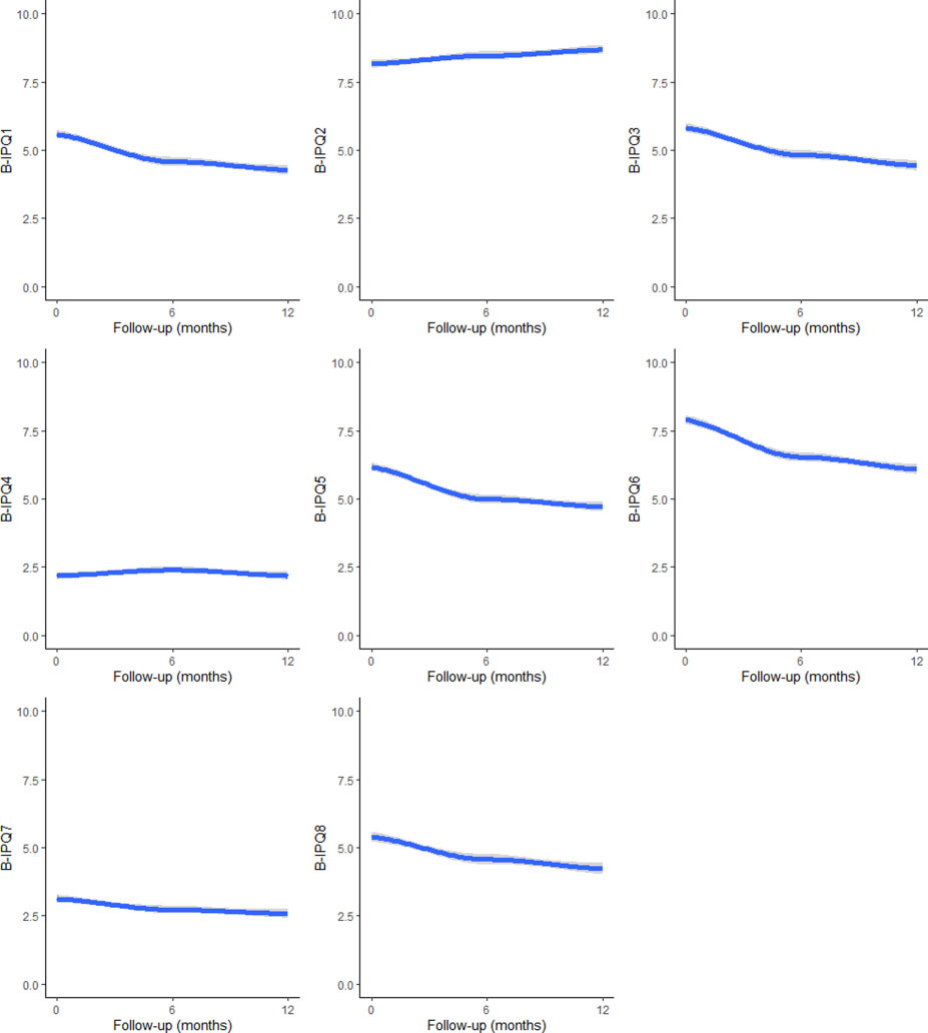
B-IPQ scores over 12 months (B-IPQ3, 4 and 7 reverse-coded) B-IPQ: Brief Illness Perception Questionnaire

In general, the illness perceptions of patients improved over 12 months (Fig. 1; Table 2). Patients rated their illness as affecting them less and they felt they had more control over their illness, were less concerned about their illness and understood their illness better. The only two B-IPQ items that did not change were B-IPQ2 and B-IPQ4, indicating that patients’ perceptions of the chronicity of their illness and the effectiveness of their treatments did not change over 12 months.

### Latent trajectories of B-IPQ scores over 12 months

**Fig. 2:**
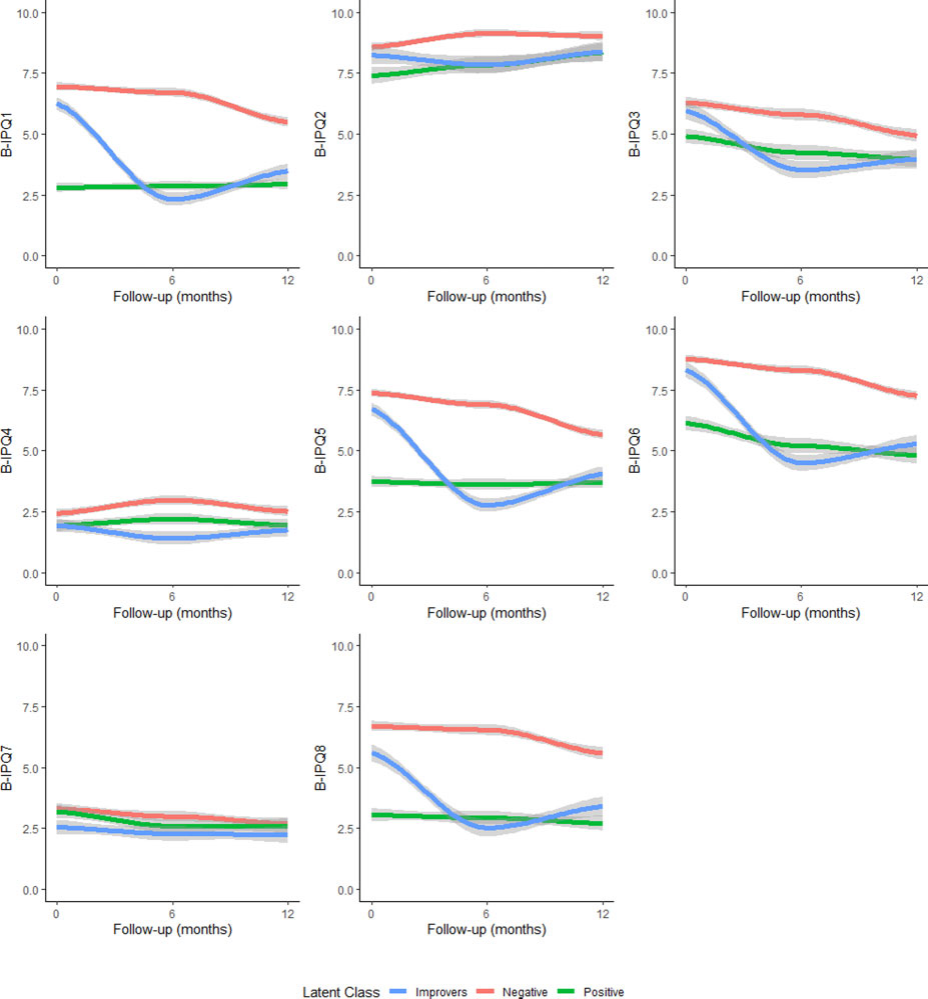
B-IPQ scores over 12 months, stratified by trajectory group B-IPQ: Brief Illness Perception Questionnaire; higher scores indicate negative illness perceptions—B-IPQ3, 4 and 7 reverse-coded

The latent class analysis identified three latent classes of B-IPQ scores over follow-up (Fig. 2; Table 2). B-IPQ items 1, 5, 6 and 8 showed the most heterogeneity, indicating that the classes were separated mainly by perceptions of the severity of their illness and the impact on patients physically and emotionally. The Negative B-IPQ trajectory group [*N* = 534 (49.1%)] had high scores on all B-IPQ items at baseline, with the majority of these items improving by approximately one unit by 12 months, the only exceptions being B-IPQ2 (longevity) and B-IPQ4 (treatment efficacy), which did not change in this group over follow-up. The Positive B-IPQ trajectory group [*N* = 322 (29.6%)] had significantly lower scores on all B-IPQ items than the Negative group, other than B-IPQ7 (illness comprehensibility), which did not differ between the trajectories. The final group comprised patients whose B-IPQ scores improved over follow-up [*N* = 231 (21.3%)]. For the majority of B-IPQ items, the Improvers group’s baseline B-IPQ scores were similar to those of the Negative trajectory group. However, by 6 months the Improvers group’s scores were similar to the Positive trajectory group’s, and this was maintained to 12 months. The only items where this was not seen were B-IPQ4 (treatment efficacy) and B-IPQ7 (illness comprehensibility), where the Improvers group had slightly better scores over follow-up than the other two groups (Table 2).

### Predictors of trajectory group membership

The results of the component of the multinomial logistic regression comparing the Positive *vs* Negative trajectory groups showed that lower baseline disability was strongly associated with being in the Positive group compared with the Negative group [relative risk ratio (RRR) 0.37 per unit increase in HAQ, 95% CI: 0.25, 0.54]. Lower pain, lower fatigue, lower depression and lower anxiety were also all independently associated with being in the Positive trajectory group compared with the Negative group (Table 3). However, baseline inflammation level (DAS28-2C) did not predict Positive *vs* Negative trajectory group membership (RRR 0.99 per unit increase in DAS28-2C, 95% CI: 0.86, 1.13).

**Table 3:** Results from multivariable multinomial logistic regression model predicting trajectory group membership from baseline variables

	Positive *vs* negative trajectory group	Improvers *vs* negative trajectory group
Baseline variable	RRR (95% CI)	RRR (95% CI)
Age (years)	1.01 (0.99, 1.03)	1.00 (0.99, 1.01)
Men *vs* women	0.72 (0.48, 1.06)	0.91 (0.63, 1.31)
Disease duration (months)	1.00 (0.97, 1.03)	1.00 (0.97, 1.03)
Smoking		
*Ex vs Never smoker*	0.96 (0.64, 1.43)	0.95 (0.66, 1.38)
*Current vs Never smoker*	1.06 (0.61, 1.81)	0.71 (0.42, 1.20)
IMD, per quintile increase	1.01 (0.88, 1.17)	1.03 (0.90, 1.17)
DAS28-2C	0.99 (0.86, 1.13)	1.13 (1.00, 1.27)
HAQ	0.37 (0.25, 0.54)	0.60 (0.43, 0.83)
Pain VAS		
*Natural scale*	0.98 (0.97, 0.99)	1.00 (0.99, 1.01)
*Standardized scale*	0.52 (0.40, 0.67)	0.97 (0.77, 1.23)
Fatigue VAS		
*Natural scale*	0.98 (0.97, 0.99)	0.99 (0.98, 1.00)
*Standardized scale*	0.60 (0.47, 0.77)	0.73 (0.58, 0.91)
HADS Anxiety	0.91 (0.85, 0.97)	0.94 (0.89, 0.99)
HADS Depression	0.91 (0.84, 0.98)	0.97 (0.91, 1.04)
RF positive *vs* negative	1.38 (0.88, 2.17)	1.13 (0.75, 1.71)

B-IPQ: Brief Illness Perception Questionnaire; DAS28-2C: 2-Component Disease Activity Score; HADS: Hospital Anxiety and Depression Scale; IMD: Index of Multiple Deprivation 2010; RRR: relative risk ratio; VAS: Visual Analogue Scale.

The key factors that predicted patients being in the Improvers group over the Negative trajectory group were lower disability (RRR 0.60 per unit increase in HAQ, 95% CI: 0.43, 0.83), fatigue (RRR 0.73 per standard deviation increase in fatigue VAS, 95% CI: 0.58, 0.91) and anxiety (RRR 0.94 per unit increase in HADS anxiety, 95% CI: 0.89, 0.99). Higher inflammation level was associated with higher odds of being in the Improvers group over the Negative group (RRR 1.13 per unit increase in DAS28-2C, 95% CI: 1.00, 1.27) (Table 3).

### Outcomes over 1 year

Disability, pain and fatigue scores were all lower in the Positive and Improvers trajectory groups compared with the Negative perceptions group, after controlling for age and gender [mean difference (95% CI), Positive *vs* Negative perceptions group: HAQ = −0.75 (−0.82, −0.67), pain VAS = −24.9 (−27.3, −22.6), fatigue VAS = −27.1 (−29.9, −24.4); Improvers *vs* Negative perceptions group: HAQ = −0.59 (−0.67, −0.50), pain VAS = −19.0 (−21.6, −16.4), fatigue VAS = −22.5 (−25.5, −19.4)].

Patients in the Positive and Improvers trajectory groups had lower DAS28-2C over follow-up, controlling for age and gender [mean difference (95% CI), Positive *vs* Negative: −0.68 (−0.84, −0.52); Improvers *vs* Negative: −0.42 (−0.60, −0.25)] (see Supplementary Table S1, available at *Rheumatology* online, for comparison of DAS28 and remission rates between groups). When controlling for time-varying inflammation score, patients in the Positive and Improvers trajectory groups still had lower disability, pain and fatigue scores compared with the Negative perceptions group [mean difference (95% CI), Positive *vs* Negative perceptions group: HAQ = −0.67 (−0.74, −0.60), pain VAS = −21.0 (−23.3, −18.7), fatigue VAS = −25.0 (−27.8, −22.2); Improvers *vs* Negative perceptions group: HAQ = −0.54 (−0.62, −0.46), pain VAS = −16.6 (−19.1, −14.1), fatigue VAS = −21.1 (−24.2, −18.1)].

## Discussion

This study illustrates how illness perceptions change in a large cohort of patients with RA over the first year of treatment with MTX. This was a cohort of patients with recent onset of symptoms. At baseline, their understanding of the long-term implications of their diagnosis would have been more limited than that of those with long-standing disease, thereby increasing their uncertainty about the long-term impact. After baseline, the majority of illness perceptions improved to a small degree in the whole sample. However, patients’ beliefs about the chronicity of RA and the efficacy of treatment did not change substantially over 12 months. Latent class analysis identified three groups of patients with distinct trajectories of illness perceptions over 12 months. One group had consistently negative illness perceptions, a second group had consistently positive illness perceptions and the third group switched from negative to positive illness perceptions in the first 6 months following initiation of treatment. Disability at baseline was strongly associated with trajectory group, as were pain, fatigue, anxiety and depression. Comparing the outcomes of the three groups showed that the Positive and Improvers groups had lower disability, pain and fatigue over 12 months compared with the Negative perception group, independent of inflammation level.

The majority of the illness perceptions for the whole cohort improved by approximately one unit over the course of 12 months. This is at odds with an analysis of 75 women with RA, which reported that patients’ illness perceptions did not change over 1 and 2 years of follow-up [31]. However, that study analysed prevalent cases of RA with a mean of 11 years since diagnosis at baseline. Potentially, illness perceptions have stabilized this long after symptom onset, as would be expected according to the underlying theory [8]. Furthermore, these patients developed RA in the 1990s, before treat-to-target strategies were widely implemented, and expectations of short-term and long-term outcomes may have been different compared with patients recruited to RAMS over the last 10 years.

Latent profile analysis has been applied to the baseline illness perceptions of a cohort of patients with RA in the past [12]. Norton *et al.* identified two groups of patients—one characterized by negative illness perceptions and one by positive illness perceptions. Assessing only the baseline data of the RAMS patients would suggest a similar class structure. However, the incorporation of longitudinal data in the current study revealed a third illness perception group, accounting for about one in five patients, that switches from negative to positive illness perceptions over the first 6 months. As illness perceptions can potentially change in the first few months after starting treatment, this potentially highlights the importance of how information is framed by clinicians when providing information at the time of diagnosis and in the early phases of the disease.

Interestingly, inflammation was not independently associated with being in the Positive trajectory group over the Negative group. Instead, patient-reported outcomes were more important in predicting group membership. Patients with higher disability, pain, fatigue, anxiety or depression were more likely to have negative illness perceptions compared with the Positive and Improvers groups. In a previous study, the inflammation components of the DAS28 have been shown to be weakly associated with illness perceptions and not associated with the psychological outcomes of patients with RA [32], and long-term observational research has illustrated a disconnect between secular changes in inflammation levels and disability as well as in other patient-reported outcomes [33–35]. This indicates the need to focus on other aspects of disease symptomology alongside inflammation in order to achieve optimal outcomes for people with RA. Higher inflammation was weakly associated with increased odds of being in the Improvers group over the Negative group. Potentially, patients with higher inflammation scores and negative perceptions at baseline noted a larger change in their inflammation level over time, as their inflammation scores had more room for change, prompting a change in the perception of their condition.

When the patient-reported outcomes of the three illness perception groups were compared over 1 year, patients in the Negative illness perception group had consistently worse outcomes, independent of longitudinal inflammation. Patients in the Positive and Improvers groups had lower disability, pain and fatigue over 1 year compared with the Negative illness perception group, holding longitudinal inflammation level constant. The 2-component DAS28 was chosen as the variable to adjust for, because we aimed to adjust for inflammation level, rather than global disease activity (i.e. the DAS28, which includes both inflammation measures and subjective components). Adjusting for the 4-component DAS28 may have ‘adjusted away’ some of the association between illness perceptions and patient-reported outcomes, as some of the components of the DAS28 will be measuring similar aspects of disease to the patient-reported outcomes (e.g. tender joint count and VAS pain).

Norton *et al.* reported that patients with positive illness perceptions had better outcomes over 6 months than patients with negative illness perceptions [12], and Van der Elst *et al.* reported that baseline illness perceptions predicted poor patient-reported outcomes, despite good control of disease (DAS28-CRP < 2.6) [36]. Furthermore, Van der Elst *et al.* also showed that changes in disease activity were associated with changes in illness perceptions over 1 year in early RA [37]. However, it is unclear whether illness perceptions shape current and future outcomes, or are a reflection of the severity of patients’ outcomes. An analysis using structural equation modelling illustrated that past and present illness perceptions form a chain that mediates the relationship between past and current functioning in patients with RA [38]. The reciprocal nature of the relationship between illness perceptions and patient-reported outcomes has also been demonstrated in other rheumatic diseases (SLE and SSc) [39]. This is in line with Leventhal *et al.*’s idea of a cyclical, dynamic relationship between illness perceptions and other outcomes, whereby the initial shock of a chronic condition alongside other factors (e.g. self-assessed health status, role identities, culture) moulds the patient’s illness perceptions. These illness perceptions influence the patient’s ability to adapt to their condition, and subsequently the success of these adaptions influences the patient’s later illness perceptions [40], suggesting that interventions should be implemented early in the disease course, before this cyclical relationship can spiral out of control or negative perceptions become embedded and fixed. This is illustrated in our data, whereby the Improvers group showed improvements in their illness perceptions in the first 6 months of follow-up after treatment initiation.

Trials in other conditions have illustrated that interventions aiming to promote positive illness perceptions and beliefs can lead to improvements in symptoms, self-management and quality of life [41–44], and studies in RA have shown that beliefs and illness perceptions are linked to adherence [45, 46]. Given the intimate link between illness perceptions and outcomes in RA, demonstrated by this and other studies, interventions to improve the illness perceptions of patients with negative illness perceptions at treatment onset may have the potential to improve long-term outcomes through increased ability to adapt, better adherence to treatment and improvements in self-management, although randomized controlled trials are required to assess the efficacy of any such interventions.

The current study has a number of strengths. To our knowledge, this is the largest prospective observational study to consider illness perceptions in RA, including over 1000 patients, resulting in precise estimates of the associations between illness perception groups and outcomes. The longitudinal nature of the study means that the evolution of patients’ illness perceptions can be described and analysed. Limitations of the study include the relatively limited data available on other important factors in Leventhal *et al.*’s self-regulation theory [40], meaning that important precipitants of illness perceptions (e.g. patients’ role identities) cannot be included in the analyses. Furthermore, education data were only available for a proportion of patients and therefore were not included in the analyses.

In conclusion, we have demonstrated that patients’ illness perceptions generally improve to a small degree in the first year following the initiation of MTX therapy. We have described three distinct illness perception trajectory groups: Positive, Negative and Improvers. The likelihood of being in the Negative illness perception group was strongly associated with worse patient-reported outcomes, and patients in the Positive and Improvers group had improved outcomes over 1 year compared with the Negative group. The observation that the beliefs held by the Improvers group became more positive over time illustrates the potential importance of identifying early opportunities to intervene with appropriate interventions to improve illness perceptions, which should be assessed using randomized controlled trials.

## Supplementary Material

keaa615_Supplementary_Data
