# Supplementary material for: Changes in the illness perceptions of patients with rheumatoid arthritis over the first year of methotrexate therapy
Source: Rheumatology (Oxford). 2020 Nov 14;60(5):2355–65. doi: 10.1093/rheumatology/keaa615 (PMC8121436; doi:10.1093/rheumatology/keaa615)
Supplement: keaa615_Supplementary_Data [file keaa615_supplementary_data.zip › rhe-20-1374-File004.docx]

| **Supplementary table S1 Comparison of the DAS28 and odds of remission between the trajectory groups** | |
| --- | --- |
| DAS28 | Mean difference (95% CI) |
| Negative | Reference |
| Positive | -1.01 (-1.14, -0.87) |
| Improvers | -0.81 (-0.96, -0.66) |
| Remission at 12 months | OR (95% CI) |
| Negative | Reference |
| Positive | 3.19 (2.16, 4.72) |
| Improvers | 2.73 (1.85, 4.02) |
| Controlling for age and gender  CI = confidence interval, DAS28 = Disease Activity Score 28, OR = Odds ratio | |

**SUPPLEMENTARY MATERIAL**

Controlling for age and gender

CI: confidence interval; DAS28: Disease Activity Score 28; OR: Odds ratio

**Supplementary figure legend**

**Supplementary figure S1 flow diagram of exclusions**

**RAMS co-investigators:** Dr A Adebajo (Barnsley Hospital NHS Foundation Trust, UK); Dr F McKenna (Central Manchester University Hospitals NHS Foundation Trust, UK); Prof M Callan (Chelsea and Westminster Hospital NHS Foundation Trust, UK); Dr S Levy (Croydon Health Services NHS Trust, UK); Dr S Knight (East Cheshire NHS Trust, UK); Dr L-S Teh (East Lancashire Hospitals NHS Trust, UK); Dr J Hamilton, Dr V Saravanan (Gateshead Health NHS Foundation Trust, UK); Dr E Williams (Hampshire Hospitals NHS Foundation Trust, UK); Dr A Gough (Harrogate and District NHS Foundation Trust, UK); Dr J Galloway, Prof D Scott (King's College Hospital NHS Foundation Trust, UK); Dr L Macphie (Lancashire Care NHS Foundation Trust, UK); Dr L Pollard (Lewisham and Greenwich NHS Trust, UK); Prof D Symmons (Macclesfield District General Hospital, UK); Prof K Hyrich (Manchester University Hospitals NHS Foundation Trust, UK); Dr T Marshall (Norfolk and Norwich University Hospitals NHS Foundation Trust, UK); Dr M Perry, Dr L Robertson, Dr N Viner (Plymouth Hospitals NHS Trust, UK); Dr A Cooper (Portsmouth Hospitals NHS Trust, UK); Dr K Ahmed (Princess Alexandra Hospital NHS Trust, UK); Dr M Davis (Royal Cornwall Hospitals NHS Trust, UK); Prof H Chinoy (Salford Royal NHS Foundation Trust, UK); Dr R Smith (Salisbury NHS Foundation Trust, UK); Dr C Mathews (South London Healthcare NHS Trust, UK); Dr C Marguerie (South Warwickshire NHS Foundation Trust, UK); Prof B Dasgupta (Southend University Hospital NHS Foundation Trust, UK); Dr S Hider, Dr S Kamath (Staffordshire and Stoke-on-Trent Partnership NHS Trust, UK); Dr D Roy (Tameside and Glossop Integrated Care NHS Foundation Trust, UK); Dr S Lane (The Ipswich Hospital NHS Trust, UK); Dr M Lee (The Newcastle upon Tyne Hospitals NHS Foundation Trust, UK); Dr S Naz (The Pennine Acute Hospitals NHS Trust, UK); Dr A Al-Ansari, Dr R Amarasena (The Robert Jones and Agnes Hunt Orthopaedic Hospital NHS Foundation Trust, UK); Dr G Smith (The Rotherham NHS Foundation Trust, UK); Dr B Quilty (The Royal Bournemouth and Christchurch Hospitals NHS Foundation Trust, UK); Dr N Viner (Torbay and South Devon NHS Foundation Trust, UK); Dr N Gullick (University Hospitals Coventry and Warwickshire NHS Trust, UK); Dr P Sanders (University Hospital of South Manchester NHS Foundation Trust, UK); Dr W Hassan (University Hospitals of Leicester NHS Trust, UK); Dr M Bukhari (University Hospitals of Morecambe Bay NHS Foundation Trust, UK); Dr E Gladston Chelliah (Wrightington, Wigan and Leigh NHS Foundation Trust, UK); Dr M Green (York Teaching Hospital NHS Foundation Trust, UK)
