# Supplementary figures and images for: Changes in the illness perceptions of patients with rheumatoid arthritis over the first year of methotrexate therapy
Source: Rheumatology (Oxford). 2020 Nov 14;60(5):2355–65. doi: 10.1093/rheumatology/keaa615 (PMC8121436; doi:10.1093/rheumatology/keaa615)

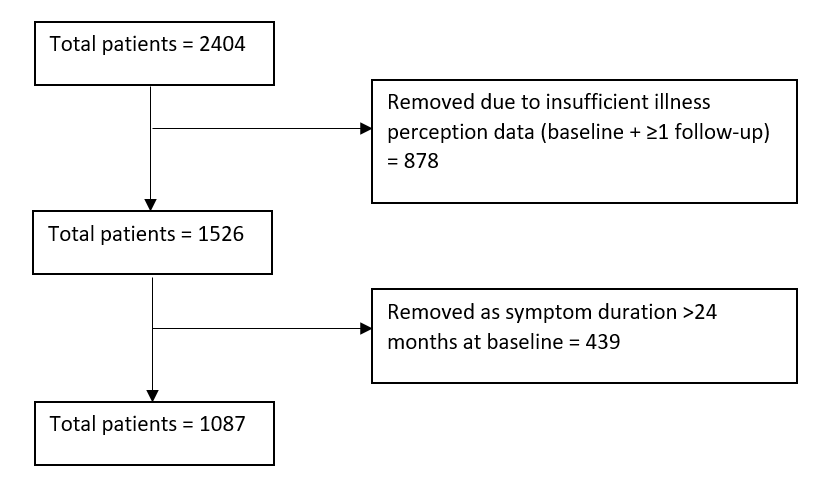

Supplement: keaa615_Supplementary_Data [file keaa615_supplementary_data.zip › rhe-20-1374-File005.tif]
